# *Chlamydia trachomatis* infection of human endometrial stromal cells induces defective decidualisation and chemokine release

**DOI:** 10.1038/s41598-017-02223-z

**Published:** 2017-05-17

**Authors:** Sevi Giakoumelou, Nick Wheelhouse, Jeremy Brown, Jean Wade, Ioannis Simitsidellis, Douglas Gibson, Philippa T. K. Saunders, Patrick Horner, Gary Entrican, Sarah E. M. Howie, Andrew W. Horne

**Affiliations:** 1 Centre for Reproductive Health, University of Edinburgh, Edinburgh, EH16 4TJ UK; 2 Moredun Research Institute, Pentlands Science Park, Bush Loan, Edinburgh, EH26 0PZ UK; 3 School of Social and Community Medicine, University of Bristol, Bristol, BS8 2BN UK; 4 The Roslin Institute and Royal (Dick) School of Veterinary Studies, University of Edinburgh, Easter Bush, Midlothian, EH25 9RG UK; 5 MRC Centre for Inflammation Research, University of Edinburgh, Edinburgh, EH16 4TJ UK

## Abstract

Miscarriage affects ~20% of pregnancies and maternal infections account for ~15% of early miscarriages. *Chlamydia trachomatis* (*Ct*) has been associated with miscarriage but the underlying mechanisms are unknown. Successful implantation requires endometrial stromal cell (ESC) decidualisation. Maintenance of pregnancy requires angiogenesis, establishment of the correct cellular milieu and trophoblast invasion, all of which involve the action of chemokines. Our objective was to determine whether *Ct* infection impacts upon ESC decidualisation and chemokine secretion. Human primary ESC were decidualised *in-vitro*, infected with *Ct* serovar E, and changes in expression of genes of interest were measured using RT-PCR, proteomic array and ELISA. We demonstrate for the first time that *Ct* can infect and proliferate in ESC. Expression of the decidualisation marker prolactin was decreased in *Ct*-infected ESC at both mRNA and protein levels. *Ct* infection altered the chemokine profile of decidualised ESC as shown by proteomic array. Chemokines CXCL12 and CXCL16, important for trophoblast invasion, were analysed further and expression was reduced in infected decidualised cells at mRNA and protein levels. Our data indicate that *Ct* infection of ESC impairs decidualisation and alters chemokine release. These findings at least partially explain how *Ct* infection could result in adverse pregnancy outcomes.

## Introduction

A miscarriage is defined as the spontaneous loss of a pregnancy during the first 24 weeks of gestation and occurs in approximately 20% of clinically recognised pregnancies^1^. Miscarriages are associated with considerable physical and psychological morbidity. Bleeding due to miscarriage can lead to haemodynamic shock and death and the emotional response to miscarriage can include depression and anxiety^1^. Approximately, 50% of early miscarriages are attributed to fetal chromosomal abnormalities, however, the underlying cause in other cases is often undefined. A number of infections have also been linked to miscarriage and infections are thought to account for 15% of early and 66% of late miscarriages (reviewed in ref. 1). Several studies have been published regarding the association of pelvic *Chlamydia trachomatis* (*Ct)* infection with miscarriage, with *Ct* prevalence ranging between 11–69% in miscarriages compared to 2–7% in healthy pregnant controls^2–6^. The mechanisms underlying this association between *Ct* infection and miscarriage are unknown, though a recent study suggests *Ct* may interfere with essential early pregnancy inflammatory processes^7^.

The development of a successful pregnancy depends upon maternal receptivity during the implantation window. This is largely established during decidualisation, the process whereby the stromal cells of the endometrium undergo structural and morphological changes to prepare for possible embryo implantation. Secretion of appropriate chemokine signals by decidual cells contributes to the recruitment of predominantly anti-inflammatory leukocyte subpopulations necessary for pregnancy maintenance^8^, and prevents recruitment of potentially damaging T lymphocytes^9^. The maternal immune response to miscarriage associated infections can have detrimental effects on pregnancy maintenance, a characteristic example of which is seen when malaria pathogens are detected in the placenta (reviewed in ref. 1). Furthermore, chemokines not only recruit and impact on immune cells but are also involved in trophoblast invasion and angiogenesis during early pregnancy^10^.

Both undifferentiated and decidualised endometrium has been shown to be altered compared to normal pregnancies^11^ in women with spontaneous miscarriage. Impaired decidualisation, measured by a reduction in the decidualisation marker prolactin (PRL) in the endometrium, has been associated with recurrent miscarriage^12^ and in rodent models decidual cell prolactin production has been shown to be critical for successful pregnancy^13^. Infection can markedly change the chemokine profile to recruit pro-inflammatory cell subsets.

It is well established that *Ct* infects endometrial epithelial cells^14–16^, however the effect of *Ct* infection on endometrial stromal cell function and decidualisation is yet undetermined and may have a role in the association of *Ct* infection with miscarriage. *Ct* is known to cause endometritis, namely inflammation of the endometrium that is often asymptomatic, in non-pregnant women^17^. Data from animal studies indicate that in mice, *C*. *abortus* induces the murine equivalent of miscarriage without fetal harm, likely due to decidual damage^18^. In cattle *C*. *psittaci* associated chronic endometritis is a recognized cause of infertility (strain now known as *Chlamydia pecorum*)^19^. To our knowledge, no study to date has identified why *Ct* can cause endometritis in women or how infection of the stromal compartment of the endometrium might alter the function of human endometrial stromal cells. We therefore aimed to determine whether *Ct* can infect human endometrial stromal cells (ESC) and examine the effect of *Ct* infection on decidualisation and chemokine secretion in an *in-vitro* model.

## Results

### *Ct* can directly infect human endometrial stromal cells (ESC)

**Figure 1:**
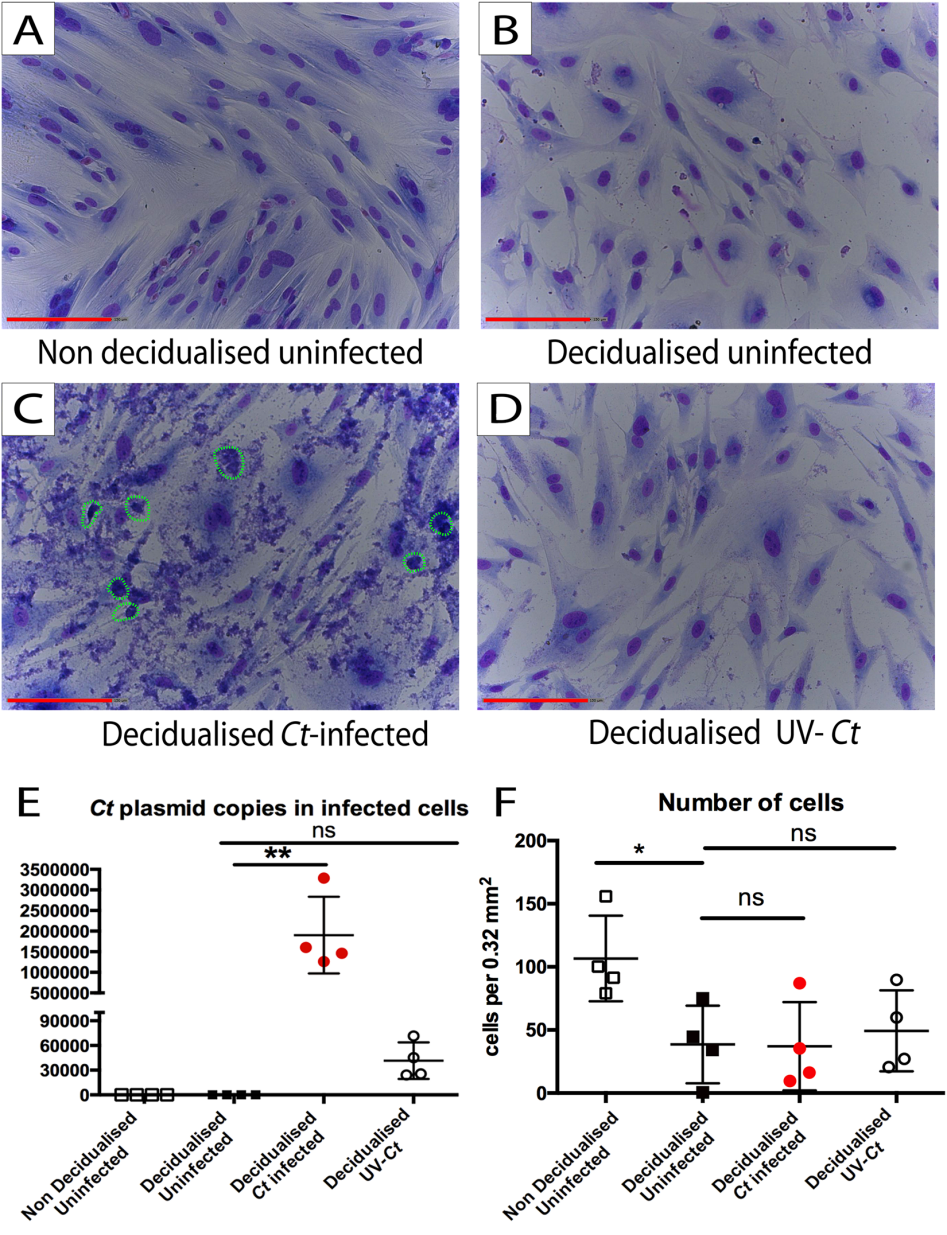
Ct infects decidualised ESC. ESC were infected with Ct MOI 2 following an *in-vitro* decidualisation protocol. Non decidualised uninfected ESC, uninfected decidualised ESC and UV-Ct treated ESC were used as controls. 48 hours post infection DNA was collected for qPCR and cells were stained with Giemsa. Cell counts were conducted in 15 fields of view per well measuring 0.32 mm^2^ each. (**A**) Non decidualised uninfected ESC were elongated and thin. (**B**) Decidualised uninfected ECS became rounder and larger compared to non decidualised ESC. (**C**) Ct infected decidualised ESC displayed signs of infection and contained inclusions that were stained purple by Giemsa stain (highlighted by green circles). (**D**) UV-Ct treated decidualised ESC did not contain chlamydial inclusions and resembled uninfected decidualised ESC in appearance. (**E**) 25.000–75.000 Ct plasmid DNA copies were detected in UV-Ct treated ESC. Ct infected ESC had a significantly higher number of 1.200.000–3.200.000 plasmid copies per sample, indicating proliferation of Ct only in infected cells (RM one-way ANOVA-Friedman’s test with Dunn’s multiple comparisons test, p = 0.0094, n = 4). (**F**) Cell counts on Giemsa stained ESC indicated that decidualised uninfected cells were significantly fewer compared to non-decidualised controls (RM one-way ANOVA-Friedman’s test with Dunn’s multiple comparisons test, p = 0.0185, n = 4). In contrast, UV-Ct treated cells and Ct infected cell numbers were similar to uninfected decidualised ESC. Scale bars equal 200 μm. Graphs show the mean and standard deviation.

Primary ESC (n = 4) were infected in 12 well plates with *Ct* serovar E at a multiplicity of infection (MOI) of 0.01, 0.1, 1, 2 and 3. No visible inclusions were present in cells at MOI 0.01 and MOI 0.1 48 hours post infection and few inclusions were observed at MOI 1 (data not shown). The non-infected ESC and ESC treated with UV-inactivated *Ct* at MOI 2, displayed no signs of infection (Fig. 1A–C) whereas visible chlamydial inclusions were seen in cells infected at MOI 2 (Fig. 1D,E). No copies of *Ct* cryptic plasmid were detected in uninfected cells, as assessed using qPCR. ESC exposed to UV-inactivated *Ct* (which still contained bacterial DNA) had 2.5 × 10^4^–7.5 × 10^4^ plasmid copies per well. *Ct* infected wells contained 1.2 × 10^6^–3.8 × 10^6^ plasmid copies indicating that significant replication had occurred (Fig. 1F). Although the number of decidualised uninfected ESC was decreased compared to non-decidualised ESC, UV-treated and *Ct* infected ESC samples contained similar numbers of cells compared to decidualised uninfected ESC (Fig. 1G). In infected wells, between 0.05–20% of ESC contained chlamydial inclusions (Supplementary Fig. S1). These conditions were used for all subsequent experiments. Cells infected at MOI 3 appeared similar to those infected at MOI 2 (equivalent to 4 × 10^5^ organisms per well, Supplementary Fig. S1).

### *Ct* infection of human ESC results in defective decidualisation

**Figure 2:**
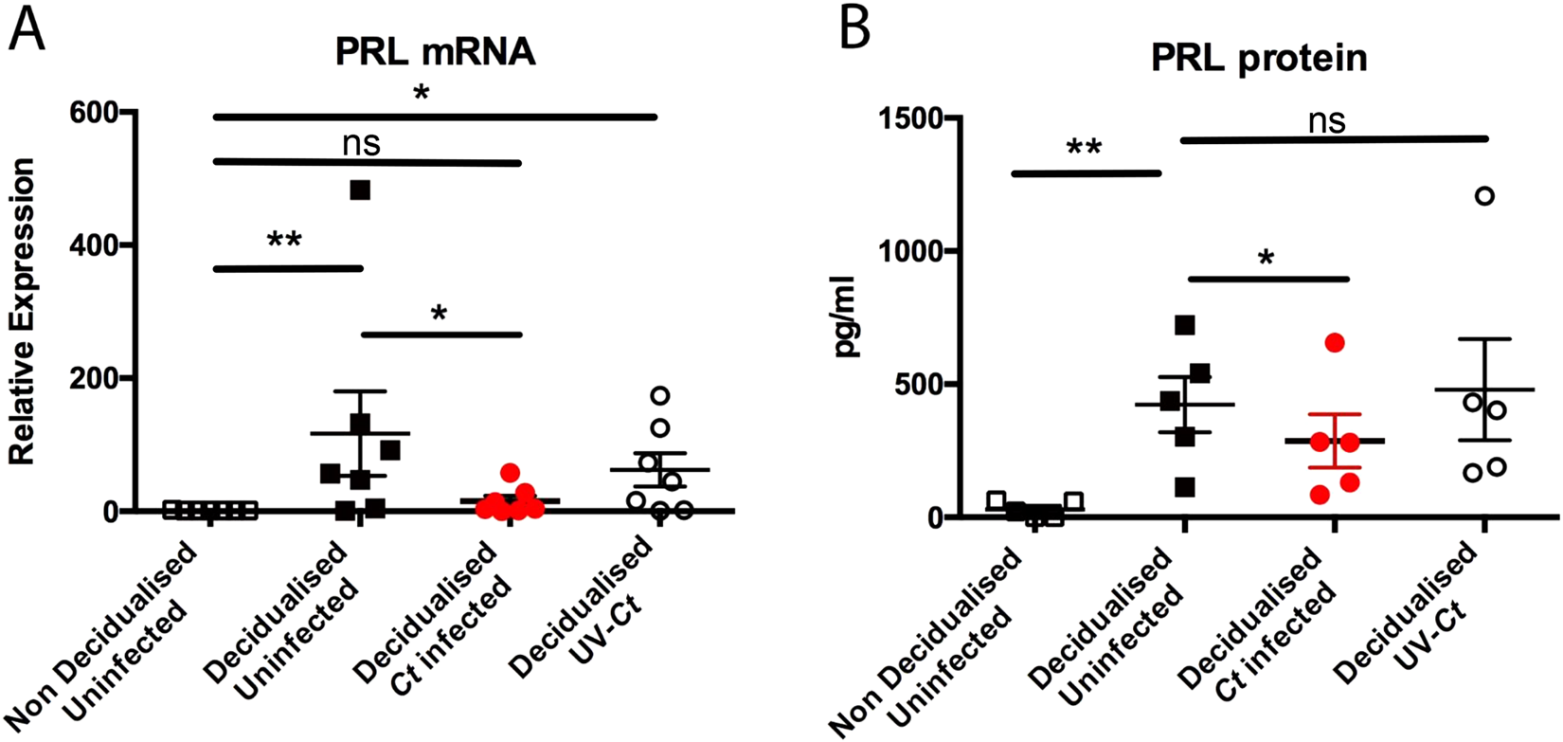
*Ct* serovar E infection of ESC results in suboptimal decidualisation of ESC. (**A**) PRL mRNA is upregulated in response to decidualisation stimulus, whereas it is downregulated in infected decidualised cells compared to uninfected controls (n = 5, One way ANOVA Friedman’s test with Dunn’s multiple comparisons test, p = 0.0009 and 0.0151 respectively). (**B**) PRL protein levels are increased in decidualised non infected cells compared to non decidualised controls as expected (n = 5, One way ANOVA Friedman’s test with Dunn’s multiple comparisons test, p = 0.0044). Reduction of PRL is observed only in cells infected by *Ct* compared to decidualised controls, indicating the adverse effect of *Ct* infection on decidualisation (n = 5, One way ANOVA Friedman’s test with Dunn’s multiple comparisons test, p = 0.028). Each circle/square represents an individual patient.

Infection with *Ct*, but not treatment with UV-inactivated *Ct*, reduced the expression of the classic decidualisation marker prolactin (PRL) at both mRNA (Fig. 2A) and protein levels (Fig. 2B) in decidualised *Ct* infected ESC. Similarly, exposure of decidualised ESC to either 200 or 100 µg/ml lipopolysaccharide (LPS) derived from either, *Escherichia coli* (*E*. *coli*) or *Salmonella enterica* serotype Minnesota (*S*. Minnesota) also failed to inhibit the of prolactin secretion (Supplementary Fig. S2). Decidualised uninfected ESC had increased levels of both PRL mRNA and protein compared to non decidualised ESC.

### *Ct-*infected human ESC have an altered chemokine profile

**Figure 3:**
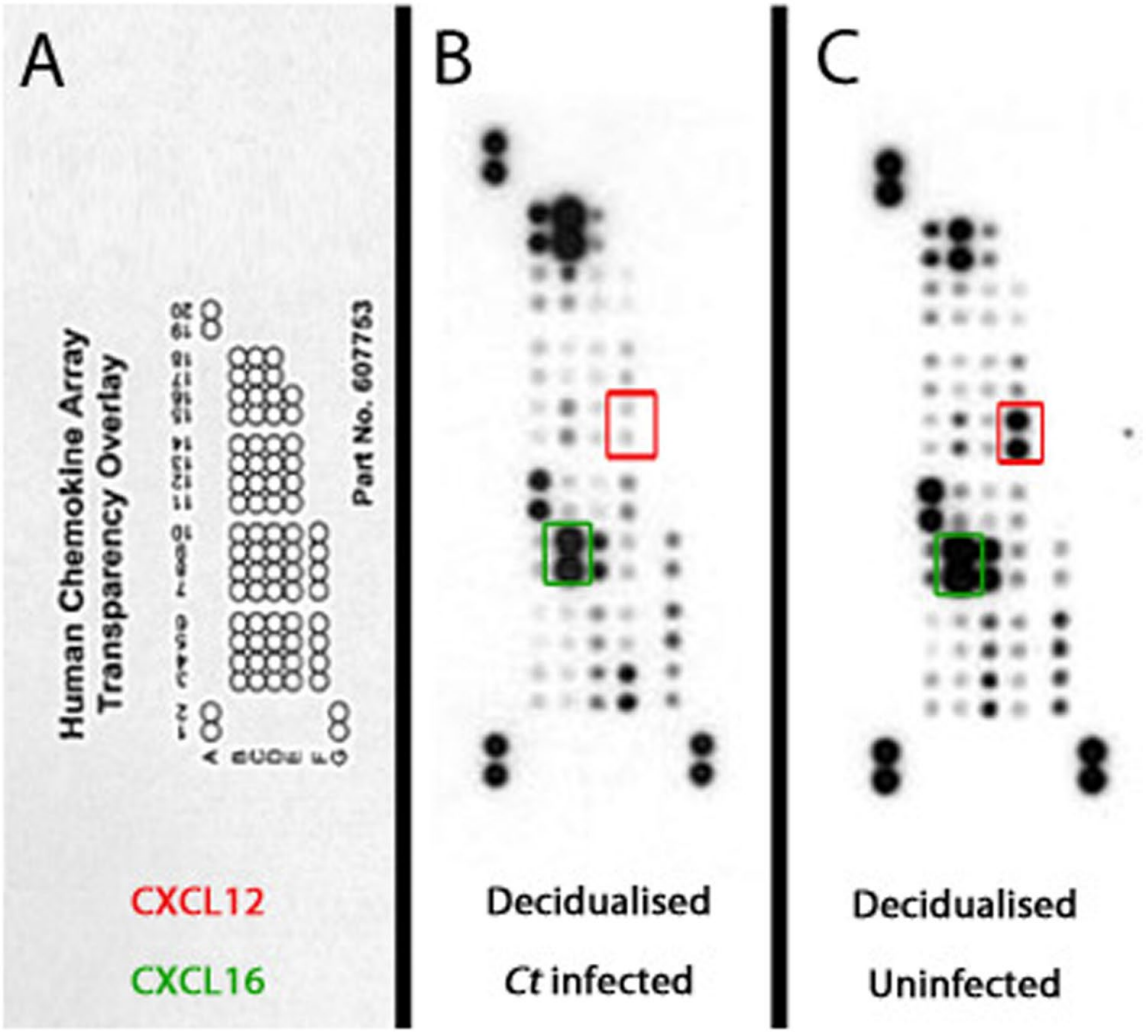
Chemokine proteomic array of 31 chemokines on pooled cell supernatants from *Ct* infected and uninfected decidualised cells. Each chemokine is represented by a dot. (**A**) *Ct* infected decidualised ESC secreted chemokine profile, demonstrating ESC secrete all 31 chemokines. (**B**) Uninfected decidualised ESC secreted chemokine profile that is altered compared to *Ct* infected ESC (n = 3).

**Figure 4:**
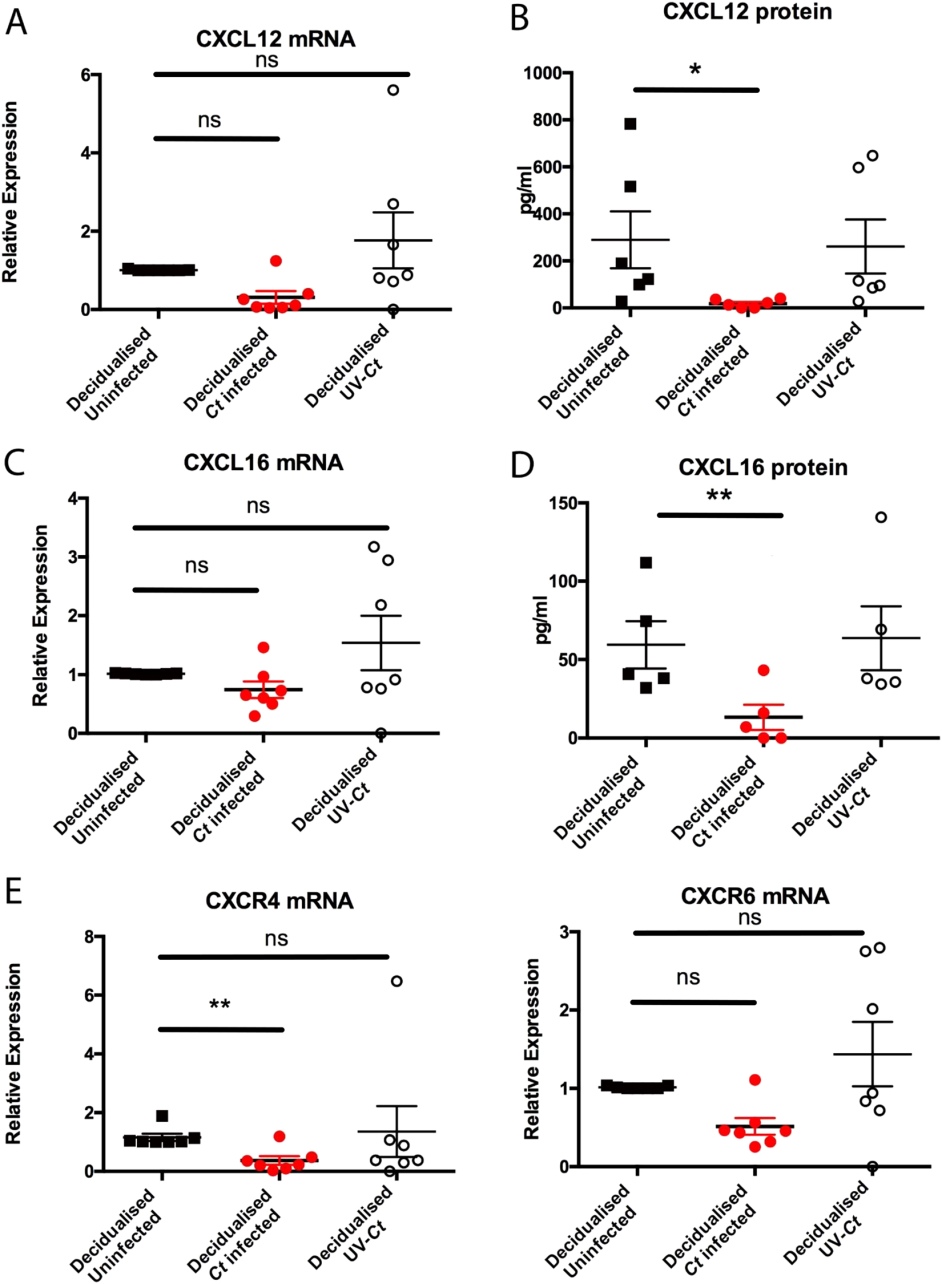
Innate immune response of decidualised ESC to *Ct* infection. The innate response was determined by measurement (at an mRNA and protein level) of trophoblast invasion-associated chemokines CXCL12 and CXCL16. (**A**,**B**) CXCL12 mRNA was not altered in response to infection, however there was a reduction in protein levels in cell supernatants (n = 4, One way ANOVA Friedman’s test with Dunn’s multiple comparisons test p = 0.0267). (**C**,**D**) Similarly, there was less secreted CXCL16 protein observed only in infected cells however no change in mRNA levels (n = 4, One way ANOVA Friedman’s test with Dunn’s multiple comparisons test, p = 0.0078). (**E**) CXR4 mRNA, receptor of CXCL12, was increased only in infected cells (n = 7, One way ANOVA Friedman’s test with Dunn’s multiple comparisons test, p = 0.0267). (**F**) CXCR6, receptor of CXCL16 does not seem to be affected by *Ct* infection in mRNA level. Each circle/square represents an individual patient.

To investigate the effect of *Ct* infection on chemokine secretion by decidualised ESC, the levels of 31 secreted chemokines were examined by proteomic array in supernatants from decidualised uninfected and infected ESC. All of the chemokines on the array were detected and a pattern of preferential expression between infected and uninfected cells was seen (Fig. 3). Supplementary Table S3 shows that four chemokines were upregulated and 17 downregulated in *Ct* infected decidualised compared to non-infected ESC (cutoff 5%). Of the chemokines known to be involved with early pregnancy, trophoblast invasion and/or genital infection CXCL12 and CXCL16 were both strongly downregulated in infected decidualised ESC, as were chemokines CCL7, CCL12 and Midkine whilst CXCL7, CXCL1 and XCL1 were strongly upregulated (see Supplementary Table S3 for further details). To validate the array results, qPCR and ELISA were used to measure changes in mRNA and protein levels of chemokines CXCL12 and CXCL16 as a result of infection with *Ct*. As seen in Fig. 4A and C, there were no significant differences in expression of mRNA encoding CXCL12 and CXCL16. In contrast, infected ESC secreted significantly less CXCL12 and CXCL16 (Fig. 4B,D). Expression of mRNA encoding CXR4 (the receptor for CXCL12) was significantly decreased in infected decidualised ESC compared to uninfected or (Fig. 4E). In contrast, expression of CXCR6 (the receptor for CXCL16) mRNA was not affected by *Ct* infection (Fig. 4F).

## Discussion

Herein, we demonstrate for the first time that *Ct* can infect and proliferate in human endometrial stromal cells (ESC) and that active infection impairs decidualisation and alters the secretion of chemokines.

To our knowledge, all previous studies have only explored the effect of *Ct* on epithelial cells of the female reproductive tract^20–22^, however as we have demonstrated here, *Ct* can also infect and affect the function of ESC. In light of this novel finding, we propose that ascending genital *Ct* infection might be a more complicated process than previously thought, as *Ct* could breach the epithelial barrier of the endometrium and infect other cell types such as stromal cells, endothelial cells or glandular epithelial cells (endometrial structure reviewed in ref. 1) causing extended inflammation. Infection with *Ct* has been reported to cause endometritis and some studies have suggested an association of chronic endometritis with miscarriage and implantation failure^23, 24^. The effect of a *Ct* infected endometrial stromal compartment on gestation-related processes, such as decidualisation, might at least partially explain infection related adverse pregnancy outcomes.

Using an *in-vitro* model, we demonstrated that *Ct* infection attenuates ESC decidualisation. Furthermore, in the current model active *Ct* infection, but not exposure to either UV-inactivated organisms or to purified *E*. *coli* or *S*. Minnesota LPS, caused a reduction in mRNA and protein levels of the widely used phenotypic decidualisation marker, PRL. PRL is a key factor in the process of decidualisation and is thought to also be involved in epithelial cell differentiation, implantation, angiogenesis, trophoblast cell growth and immune regulation during early pregnancy^25^. We believe that this observation is important because a reduction of endometrial PRL has been linked recently to recurrent miscarriage^12^.

Furthermore, we show that *Ct* infection changes the chemokine secretion profile in decidualised ESC. Interestingly, we observed a reduction in the levels of CXCL12 and CXCL16. The reduction in protein expression in the culture supernatants cannot be attributed to increased receptor-ligand interactions as the receptor for neither chemokine showed increased expression. We believe that this may have relevance to the role of *Ct* in miscarriage because these cytokines are known to promote trophoblast migration^26–28^. It is possible that that infection could therefore lead to defective trophoblast invasion due to lack of essential attractant signalling molecules secreted from maternal decidua, such as CXCL12 and CXCL16.

Our findings are also important because decidualised endometrium from spontaneous and recurrent miscarriages have been reported to have different immune cell profiles compared to viable pregnancies, including increased levels of uterine natural killer cells (uNK) and macrophages^29, 30^. Dysregulated chemokines due to *Ct* infection, as indicated in our array, could therefore also impact on the population of immune cells at the feto-maternal interface by altering immune cell recruitment.

In summary, our data suggest a novel mechanism through which infection leads to defective endometrial stromal cell decidualisation, resulting in an altered immune response that could impact upon trophoblast migration and immune cell recruitment. Future work to clarify the potential role of *Ct* and other bacterial infections upon trophoblast invasion via CXCL12 and CXCL16 dysregulation and immune cell recruitment in the endometrium due to altered chemokine profile could further our understanding of this potential mechanism of infection associated miscarriage.

## Materials and Methods

### Subjects

Ethical approval for this study was obtained from the Lothian Research Ethics Committee (LREC10/S1402/59). Informed written consent obtained from all patients and all of the methods were carried out in accordance with the approved guidelines. Human endometrial stromal cells (ESC) were collected after informed written consent from women undergoing hysterectomy for benign gynaecological conditions. Only samples from women in the proliferative phase of the menstrual cycle (cycle staging determined by the last menstrual period and measurement of serum estradiol and progesterone levels) were selected for further experiments. The tissue was dissociated using enzymatic digestion with 1 mg/ml of collagenase type IV for two hours at 37 °C, (Sigma C5138), followed by mechanical breakdown using a 70 μm and subsequently 40 μm filter (Falcon, Corning, Cat. Nos 352350 and 352340 respectively). Single cells were plated in RPMI 1640 media (Sigma, Cat. No. R0883) supplemented with 10% heat inactivated foetal calf serum (HIFCS, Gibco, Cat. No. 10082139), 1% L-glutamine (Sigma, Cat. No. G-7513) and 1% Penicillin/Streptomycin (Sigma, Cat. No. P4333).

### Primary human endometrial stromal cell (ESC) culture

Prior to any treatments, cells were transferred to phenol red-free RPMI 1640 (Sigma, Cat. No. R7509) containing 10% charcoal stripped fetal calf serum (CSFCS, prepared in house from FCS), 1% L-glutamine and 0.5% gentamycin (Sigma, Cat. No. G1272) for 48 hours prior to use. The antibiotic gentamycin was used instead of the standard penicillin/streptomycin regimen, because it does not inhibit *Ct* growth^31^. Cells were maintained at 37 °C and 5% CO2. Treatments were completed in duplicate per experiment and were repeated on 4 (for initial chlamydial growth experiments) or 6 (prolactin/chemokine expression) occasions using cells derived from different patients on each occasion.

### *In-vitro* decidualisation

To assess the effects of chlamydial infection on the decidualisation of primary endometrial stromal cells, an *in-vitro* decidualisation method previously described was used^32^. Cells were trypsinised, spun for 3.5 minutes at 800 rpm and resuspended phenol red-free RPMI 1640 with 10% CSFCS. They were then plated at 10^5^ cells per well of a 12-well plate for a minimum of 24 hours. Before the decidualisation protocol commenced, cells were serum starved in 2% CSFCS medium for 24 hours, supplemented as previously described. The cells were treated with 10–6 M progesterone (Sigma, Cat. No. P0130) and 8-Bromo-adenosine 3′-5′-cyclic monophosphate (cAMP, Sigma, Cat. No. A9501) at a final concentration of 0.1 mg/ml. The medium was changed every 48 hours over a six-day period (Supplementary Table S4).

### Ct elementary bodies (EBs) growth and purification *Chlamydia trachomatis*

(Ct) serotype E stock was produced in HEp2 cells using well established protocols. Sub-confluent flasks of HEp2’s were inoculated with Ct. Infected cells were cultured for 48–72 hours in Iscove’s Modified Dulbecco’s Medium (IMDM, Life Technologies, Paisley, UK) supplemented with 2% heat inactivated fetal calf serum (FBS) and 1 µg/ml cycloheximide (Sigma, Cat. No. C4859) (PAA laboratories Ltd, Yeovil, Somerset, UK), until high numbers of mature inclusions were observed by optical microscopy. The cell monolayers were disrupted with glass beads and the medium containing cell debris was briefly sonicated (Vibracell, Sonics & Materials, Connecticut, USA) and centrifuged at 50 × g for 5 minutes at 4 °C to remove intact cells. The supernatant was removed and centrifuged at 12,000 × g using a J-LITE JLA-16.250 rotor (Beckman Coulter Ltd. High Wycombe, UK). The pellet was resuspended by sonication in Tris/KCL and 25 ml of inoculum were layered onto 10 ml 40% Gastrografin® (Bayer plc, Berkshire, UK) and centrifuged at 20,000 rpm at 4 °C for 45 minutes SW 32Ti rotor on Optima L-90K ultracentrifuge (Beckman Coulter Ltd). The pellet was re-suspended into 1 ml Tris/KCL and layered onto discontinuous gradient (54%, 44%, 34% Gastrografin®) and centrifuged at 20, 000 rpm at 4 °C for two hours. The interface between the 44% and 54% layers, containing the EBs was carefully removed. The EBs were washed by resuspension in 10 ml Tris KCL and centrifugation at 20, 000 rpm at 4 °C for a further 45 minutes, to remove residual traces of Gastrografin®. The final pellet was resuspended by sonication in 10 ml of Chlamydia Transport Medium (218 mM Sucrose, 3.76 mM KH2P04, 7.1 mM K2HP04, 4.9 mM L-glutamic acid, 10% FBS, 0.05 mg/ml Gentamycin, 0.1 mg/ml Streptomycin, 150 U/ml Nystatin). and the aliquots stored at −80 °C.

### UV inactivation of Ct elementary bodies

To UV inactivate *Ct* EB’s, 500 μl of inoculum was exposed to 2MJ UV-C. The successful inactivation of *Ct* was confirmed by cell culture of HeLa cells with UV inactivated bacteria for 96 hours without the presence of any inclusions.

### Titration of Ct stock

To determine the titre of the *Ct* stock, HEp2 cells were plated at 10^5^ per well of 8 well glass chamber slides, infected with serial tenfold dilutions of *Ct* inoculum (Multiplicity of infection [MOI] 1–1 × 10^−8^) and cultured for 48 hours. The cells were fixed in ice-cold acetone for 5 minutes, left to air dry and stored at −20 °C. After thawing, the slides were rehydrated in PBS and incubated with the primary antibody (*C*. *abortus* LPS, Santa Cruz Biotechnology, Cat. No. 13/4) for 1 hour at room temperature. Following PBS washes, the slides were incubated with a fluorescein isothiocyanate (FITC)-conjugated anti-mouse antibody (Sigma) for 60 minutes at room temperature in a light-tight humidity chamber. Slides were mounted using ProLong® Gold Antifade Mountant with DAPI (lifeTechonologies, Cat. No. P36930). The total number of inclusions per well was counted at serial dilutions 10^−5^–10^−7^ and the titre of stock was calculated.

### *Ct* infection of decidualised ESC

ESC were seeded at 12-well plates and decidualised as described above. On day 6, ESC were infected with *Ct* MOI 0.01, 0.1, 1, 2 and 3 or UV-*Ct* at the same MOI for 48 hours prior to sample collection. To determine whether decidualisation of ESC was affected by exposure to lipopolysaccharide (LPS) from other bacterial species, decidualised ESC isolated from a further five subjects were exposed to LPS from either *E*. *coli* or *S*. Minnesota (both Invivogen) (each at 200 μg/ml and 100 μg/ml) for 48 hours prior to sample collection.

### RNA extraction, quantification and quality assessment

To assess changes in expression levels of genes of interest, total RNA extraction was performed Using the RNeasy® Micro kit (Qiagen, Crawley, UK, Cat. No. 74004) following the manufacturer’s protocol.

### Real time PCR

All reactions were carried out in 384 well plates at a final reaction volume of 10 μl per sample, as shown in Supplementary Table S5. The samples were mixed by repeat pipetting and centrifuged in a mini plate spinner (Applied Biosystems, Warrington, UK) at 1000 g for 20 seconds to remove any bubbles. A standard curve of cDNA made from standardised placenta RNA (Ambion, Cat. No. AM7950) was used on every plate to ensure comparability among all plates. All primers were predesigned KiCqStart® SYBR® Green Primers (Cat. No. KSPQ12012).

PCR conditions were an initial step of 3 min at 95 °C, followed by 40 cycles of 5 s at 95 °C, 15 s at 60 °C and a final disassociation step consisting of 15 s at 95 °C, 15 s at 60 °C and finally 15 s at 95 °C.

### DNA extraction from ESC

To extract DNA from infected cells, the MagMAX™ Total Nucleic Acid Isolation Kit (Ambion, Cat. No. AM1840) was used according to the manufacturers’ protocol.

### Ct plasmid copy assay

To assess whether *Ct* could proliferate in ESC the ‘*Chlamydia trachomatis* Genesig Standard Kit’ (PrimerDesign, Cat. No Path-C.trachomatis-standard) was used. A standard curve of known concentration provided by the kit was used to absolutely quantify *Ct* plasmid copy numbers in DNA extracts from ESC cells using qPCR.

### Human Chemokine Array

Proteome Profiler™ Human Chemokine Array Kit (R&D Systems, Abingdon, UK, Cat. No. ARY017) was used. Samples were pooled supernatants from ESC cultures and the manufacturer’s protocol was followed without deviation. The data are expressed as percentage of positive control of each membrane and pixel density was measured using ImageJ.

### Enzyme-linked Immunoabsorbant Assay (ELISA)

The sandwich ELISA was used to detect PRL (R&D Systems, Cat. No. DY682) protein concentrations as per manufacturer’s instructions. Sample concentrations were calculated using MasterPlex® QT (Hitachi) software.

### Magnetic Luminex®Screening Assay

The Luminex®Screening Assay (R&D Systems, Cat. No. LXSAHM) was used to detect CXCL12 and CXCL16 as per manufacturer’s instructions.

### Statistical analysis

Statistical analysis was carried out using GraphPad Prism 6. Friedman’s non parametric test was used for all sample sets. The “n” number corresponds to individual patients.

## Electronic supplementary material

Supplementary figures and tables
