# Supplementary material for: Chlamydia trachomatis infection of human endometrial stromal cells induces defective decidualisation and chemokine release
Source: Sci Rep. 2017 May 17;7:2001. doi: 10.1038/s41598-017-02223-z (PMC5435679; doi:10.1038/s41598-017-02223-z)
Supplement: Supplementary file 1 — Supplementary figures and tables [file 41598_2017_2223_MOESM1_ESM.pdf]

Chlamydia trachomatis **infection of human endometrial stromal cells induces defective decidualisation and chemokine release.**

Author list:
Sevi Giakoumelou[1], Nick Wheelhouse[2], Jeremy Brown[1], Jean Wade[1], Ioannis Simitsidellis[1], Douglas Gibson[1], Philippa TK Saunders[1], Patrick Horner[3], Gary Entrican[4], Sarah EM Howie[5] and Andrew W Horne[1*].

Affiliations:
1 Centre for Reproductive Health, University of Edinburgh, Edinburgh, EH16 4TJ, UK
2 Moredun Research Institute, Pentlands Science Park, Bush Loan, Edinburgh, EH26 0PZ, UK
3 School of Social and Community Medicine, University of Bristol, BS8 2BN, UK
4 Moredun Research Institute, Pentlands Science Park, Bush Loan, Edinburgh, EH26 0PZ and The Roslin Institute and Royal (Dick) School of Veterinary Studies, University of Edinburgh, Easter Bush, Midlothian EH25 9RG, UK
5 MRC Centre for Inflammation Research, University of Edinburgh, Edinburgh, EH16 4TJ, UK

Supplementary figures and tables

Supplementary figure and table legends

Supplementary Figure S1: hESC infected with *Ct* at an estimated multiplicity of infection (MOI) 1, 2 and 3. In infected wells, between 0.05% (at an MOI 1) and 20% (at either an MOI of 2 or 3) of ESC contained chlamydial inclusions. No differences were observed in the numbers of inclusions in cells from two different patients. An MOI 2 was used for all subsequent experiments. Scalebars equal 500μm.

Supplementary Table S2. Proteomic chemokine array results (measured in percentage of positive control of each membrane) and information for the role of each chemokine in decidualisation, early pregnancy/trophoblast invasion, miscarriage and *Ct* infection.

Supplementary Figure S3: Prolactin expression and release remains unaffected by *e*xposure of decidualised ESC to LPS. a. PRL mRNA remains unaffected by exposure of decidualised ESC to either rough LPS (rLPS; derived from *S. minnesota*) or smooth LPS (sLPS; derived from *E. coli*) (n=5, One way ANOVA Friedman's test with Dunn's multiple comparisons test) b. PRL protein levels remain unaffected by exposure to LPS (n=5, One way ANOVA Friedman's test with Dunn's multiple comparisons test).

Supplementary Table S4: Decidualisation and infection protocol for human endometrial stromal cells (ESC). Treatments of progesterone (P4) and cyclic-AMP (cAMP) were administered in the cell media on days 1, 3 and 5. On day 6 the cells were infected with *C. trachomatis* or vehicle control and 48 hours later samples were collected.

Supplementary Table S5: qPCR sample using SYBR green

Supplementary Figure S1

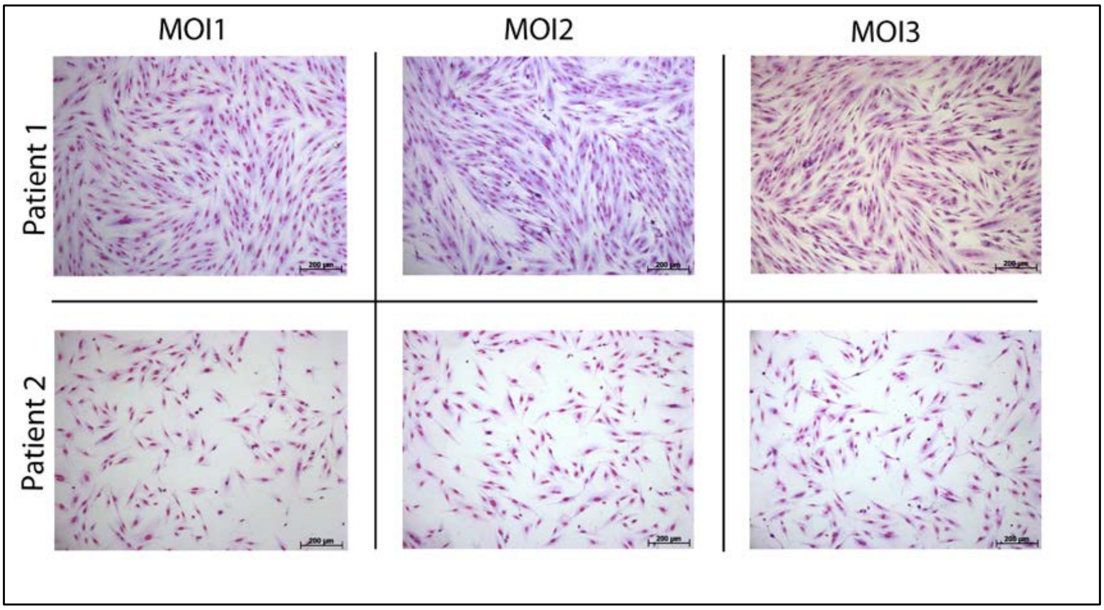

Supplementary Figure S2

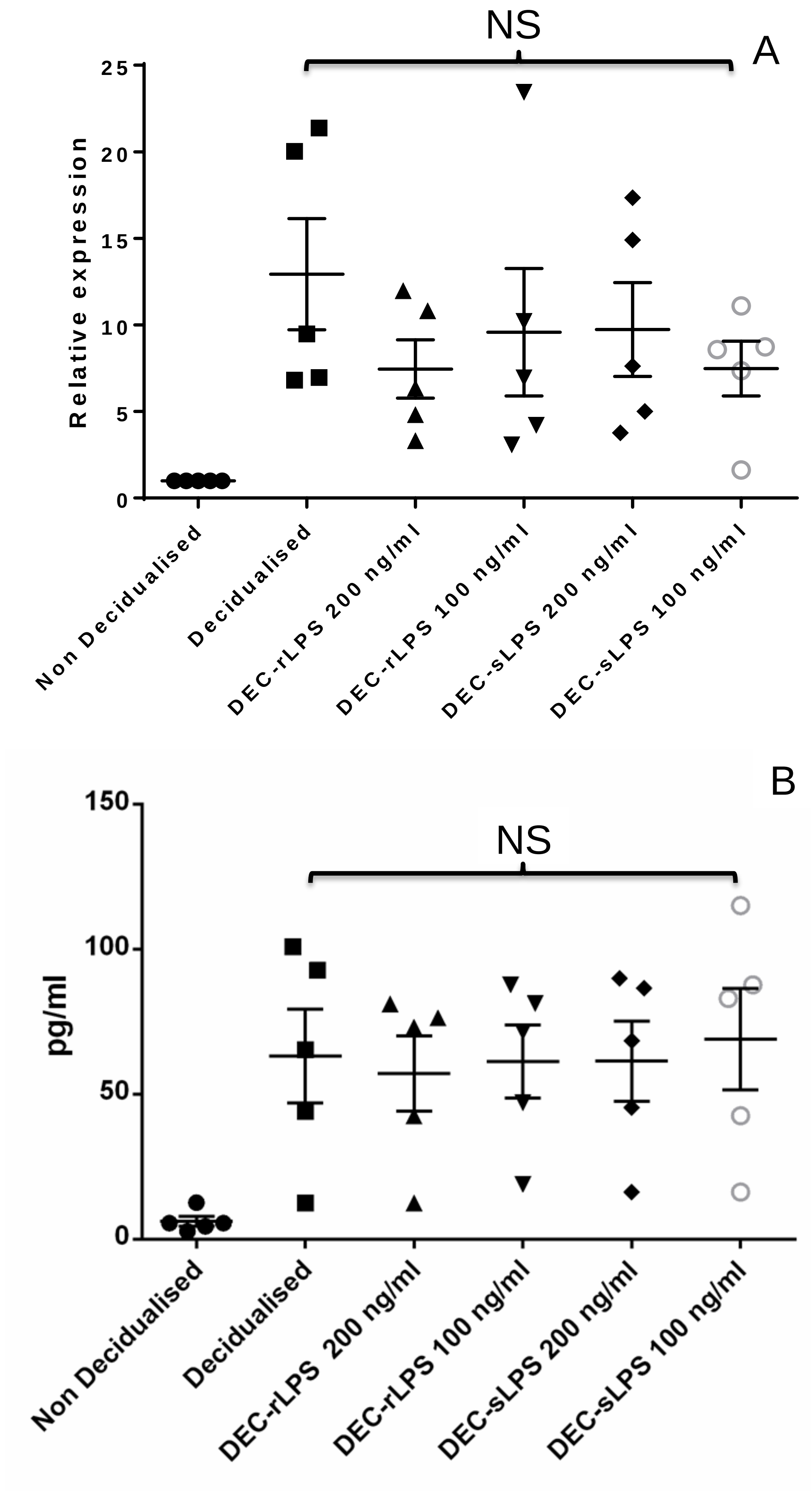

Supplementary Table S3

Gene	Decidualised *Ct* infected % of positive control	Decidualised uninfected % of positive control	Δ % of positive control	Role in decidualisation/early pregnancy/miscarriage/association with *Ct* in humans
6Ckine/ CCL21/ Exodus-2	14.90	19.43	-4.53	mRNA detected in the endometrium throughout the menstrual cycle and increased in early pregnancy (GCID:GC09M034709). No information on miscarriage and association with *Ct* infection.
CCL28/ MEC	6.42	7.78	-1.36	Detected by immunohistochemistry in decidualised hESC and first trimester decidua, was shown to induce apoptosis in hESC and its receptors were increased in hESC from miscarriage compared to controls[1]. No information on association with *Ct* infection.
CXCL16 / SRPSOX	24.59	53.31	-28.72	Human trophoblasts recruited T lymphocytes and monocytes into decidua by secretion of CXCL16 in early pregnancy [2]. CXCL16 also induces invasion and proliferation of first-trimester human trophoblast cells [3]. No information on decidualisation, miscarriage or association with *Ct* infection.
Chemerin /TIG-2/ RARRES 2	91.66	99.69	-8.03	Chemerin serum concentration was significantly higher in pregnant women in the 3rd trimester than in non-pregnant and pregnant women in the 1st trimester, whilst no differences were observed during the menstrual cycle [4]. No information on miscarriage or association with *Ct* infection.
ENA-78/ CXCL5	10.67	14.48	-3.82	Elevated ENA-78 levels were associated with increased risk of miscarriage [5]. mRNA levels were increased in trachoma patients [6]. No information on decidualisation.
Eotaxin-3/ CCL2	16.52	23.75	-7.23	IL-33 enhances proliferation and invasiveness of ESC by up-regulation of CCL2 and its receptor CCR2[7].

				No association between maternal circulation levels during the first trimester of CCL2 and miscarriage[8].<br>No information on association with *Ct* infection.
Fractalkine/ CX3CL1 / Neurotactin	28.68	30.31	-1.63	Is expressed in maternal decidua and promotes human trophoblast invasion [9].<br>No association between maternal circulation levels during the first trimester of CCL2 and miscarriage[8].<br>No information on association with *Ct* infection.
GROα/ CXCL1	94.17	66.23	27.94	Expressed in early pregnancy decidua (GCID:GC04P073869).<br>Upregulated in women with antiphospholipid antibodies inducing a placental inflammatory response via the TLR-4/MyD88 pathway, which in turn compromises trophoblast survival[10].<br>Associated with asymptomatic *Ct* infection in men [11].
HCC-1/ CCL14/ HCC-3	10.22	15.23	-5.01	Promotes human trophoblast invasion [9].<br>No information on miscarriage or association with *Ct* infection.
I-309/ CCL1/ TCA3	10.82	18.54	-7.72	No information on decidualisation, early pregnancy, miscarriage or association with *Ct* infection.
IL-8/CXCL8	98.02	100.15	-2.13	Promotes trophoblast invasion [12].<br>Upregulated in women with antiphospholipid antibodies inducing a placental inflammatory response via the TLR-4/MyD88 pathway, which in turn compromises trophoblast survival[10].<br>No association of serum levels with miscarriage (Whitcomb et al., 2007).<br>Induced in response to *Ct* infection [13].
IL-16/ LCF	20.71	36.59	-15.88	Expressed in decidua during the implantation window (GCID:GC15P081159).<br>Induced in response to *Ct* infection in cell lines [14].<br>No information on miscarriage.
IP-10/ CXCL10	36.13	44.75	-8.62	Increased in decidua during the implantation window [15]. Also reported to be increased in women with *Ct* associated infertility [16].<br>No information on miscarriage.
I-TAC/ CXCL11	8.70	13.58	-4.88	Increased levels in euthyroid women with autoimmune thyroiditis with recurrent miscarriage and compared to diabetics with successful pregnancy [17].

				Secreted by progesterone primed primary endocervical epithelial cells infected with *Ct* [18]. No information on decidualisation or early pregnancy.
Lympho tactin/ XCL1/ Lptn/ ATAC/ SCM-1α	46.07	23.09	22.98	Upregulated during implantation window [19]. No information on miscarriage or association with *Ct* infection.
MCP-1/ CCL2/ MCAF	101.83	99.08	2.75	Localised in glandular epithelium and endothelial cells, not secreted by blastocyst during early implantation winodow (GCID: GC17P034255). Upregulated in women with antiphospholipid antibodies inducing a placental inflammatory response via the TLR-4/MyD88 pathway, which in turn compromises trophoblast survival [10]. Elevated mRNA due to *Ct* infection [20].
MCP-3/ CCL7	38.34	61.95	-23.61	Detected in plasma of women during 1st trimester of pregnancy but no difference between miscarriage cases and controls [8]. Does not induce trophoblast migration [9]. Not detected in sera of infertile women with past *Ct* infection [21].
MDC/ CCL22/ STCP-1/ ABCD-1	31.95	46.88	-14.93	Detected on maternal stromal cells and increased in placentae from miscarriage cases [22]. No information on association with *Ct* infection.
MDK/M K/ Midkine	76.92	97.66	-20.75	No information on decidualisation, early pregnancy, miscarriage or association with *Ct* infection.
MIG/ CXCL9	10.27	17.56	-7.29	Increased levels in euthyroid women with autoimmune thyroiditis with recurrent miscarriage compared to diabetics with successful pregnancies[17]. Associated with asymptomatic *Ct* infection in men [11]. No information on decidualisation or early pregnancy.
MIP-1α/β / CCL3/ CCL4	10.44	18.14	-7.70	Shown to be secreted by endometrial stromal cells in response to hCG [23]. Promotes trophoblast migration [9]. No association of serum levels with miscarriage [5]. Levels were not affected by *Ct* infection [24].

MIP-1δ/ CCL15/ Leukotactin 1/ MIP-5/ HCC-2	10.74	16.56	-5.82	No information on decidualisation, early pregnancy, miscarriage or association with *Ct* infection.
MIP-3α/ CCL20/ LARC/ Exodus-1	14.12	14.36	-0.24	Was shown to be increased due to *Ct* infection [25].<br>No information on decidualisation, early pregnancy or miscarriage.
MIP-3β/ CCL19/ ELC/ Exodus-3	36.15	37.03	-0.88	No information on decidualisation, early pregnancy, miscarriage or association with *Ct* infection.
NAP-2/ CXCL7/ CTAP III	78.93	18.09	60.84	No information on decidualisation, early pregnancy, miscarriage or association with *Ct* infection.
PARC/ CCL18/ MIP-4/ AMAC-1	15.85	25.42	-9.57	Increased in trachoma patients in response to Il-17 [6].<br>No information on decidualisation, early pregnancy or miscarriage.
PF4/ CXCL4	19.27	31.85	-12.57	No information on decidualisation, early pregnancy, miscarriage or association with *Ct* infection.
RANTES / CCL5/ SISd	34.42	25.36	9.07	Localised in endometrial stromal and endothelial cells during the implantation window and is not produced by the trophoblast (GCID:GC17M035871).<br>No association of serum levels with miscarriage [5].
SDF-1/ CXCL12 / PBSF	15.12	94.86	-79.75	Associated with trophoblast invasion and implantation in primary stromal cells [26,27]. There is also a study showing inhibition of CXCL12 due to cytomegalovirus infection that resulted in impaired migration and invasion of human extravillous cytotrophoblasts [28].<br>Levels not altered in semen of men with asymptomatic *Ct* infection [11]. No information on association with miscarriage.
TARC/ CCL17	17.75	30.25	-12.50	Induces trophoblast migration [29].<br>No information on decidualisation, miscarriage or association with *Ct* infection.
VCC-1/ CXCL17 / DMC	6.77	10.00	-3.23	No information on decidualisation, early pregnancy, miscarriage or association with *Ct* infection.

Supplementary Table S4

	**Day 1**	**Day 3**	**Day 5**	**Day 6**	**Day 8**
**1° ESC/ SHT290**	P4/cAMP treatment	P4/cAMP treatment	P4/cAMP treatment	*Ct*/mock infection	Sample collection

Supplementary Table S5

Stock	Final concentration	Volume per 10µl
2x Brilliant III SYBR Mix	1X	5µl
Primer pair	10µM	0.5µl
Diluted Reference Dye		0.15 µl
Nuclease Free H_2O		3.35 µl
cDNA	2.5ng	1µl

Bibliography

1. Sun, C. *et al.* Chemokine CCL28 induces apoptosis of decidual stromal cells via binding CCR3/CCR10 in human spontaneous abortion. *Mol. Hum. Reprod.* **19,** 676–686 (2013).

2. Huang, Y., Zhu, X.-Y., Du, M.-R. & Li, D.-J. Human Trophoblasts Recruited T Lymphocytes and Monocytes into Decidua by Secretion of Chemokine CXCL16 and Interaction with CXCR6 in the First-Trimester Pregnancy. *J. Immunol.* **180,** 2367–2375 (2008).

3. Huang, Y. *et al.* Chemokine CXCL16, a scavenger receptor, induces proliferation and invasion of first-trimester human trophoblast cells in an autocrine manner. *Hum. Reprod.* **21,** 1083–91 (2006).

4. Garces, M. F. *et al.* Serum chemerin levels during normal human pregnancy. *Peptides* **42,** 138–143 (2013).

5. Whitcomb, B. W. *et al.* Circulating chemokine levels and miscarriage. *Am. J. Epidemiol.* **166,** 323–31 (2007).

6. Burton, M. J. *et al.* Active trachoma is associated with increased conjunctival expression of IL17A and profibrotic cytokines. *Infect. Immun.* **79,** 4977–83 (2011).

7. Hu, W.-T. *et al.* IL-33 enhances proliferation and invasiveness of decidual stromal cells by up-regulation of CCL2/CCR2 via NF-κB and ERK1/2 signaling. *Mol. Hum. Reprod.* **20,** 358–72 (2014).

8. Hannan, N. J., Bambang, K., Kaitu'u-Lino, T. J., Konje, J. C. & Tong, S. A bioplex analysis of cytokines and chemokines in first trimester maternal plasma to screen for predictors of miscarriage. *PLoS One* **9,** e93320 (2014).

9. Hannan, N. J., Jones, R. L., White, C. A. & Salamonsen, L. A. The chemokines, CX3CL1, CCL14, and CCL4, promote human trophoblast migration at the feto-maternal interface. *Biol. Reprod.* **74,** 896–904 (2006).

10. Mulla, M. J. *et al.* Antiphospholipid antibodies induce a pro-inflammatory response in first trimester trophoblast via the TLR4/MyD88 pathway. *Am. J. Reprod. Immunol.* **62,** 96–111 (2009).

11. Hakimi, H., Zainodini, N., Khorramdelazad, H., Kazemi Arababadi, M. & Hassanshahi, G. Seminal Levels of Pro-inflammatory (CXCL1, CXCL9, CXCL10) and Homeostatic (CXCL12) Chemokines in Men With Asymptomatic *Chlamydia trachomatis* Infection. *Jundishapur J Microbiol* **7,** e11152 (2014).

12. Jovanović, M., Stefanoska, I., Radojčić, L. & Vićovac, L. Interleukin-8 (CXCL8) stimulates trophoblast cell migration and invasion by increasing levels of matrix metalloproteinase (MMP)2 and MMP9 and integrins α5 and β1. *Reproduction* **139,** 789–798 (2010).

13. Natividad, A. *et al.* Innate immunity in ocular *Chlamydia trachomatis* infection: contribution of IL8 and CSF2 gene variants to risk of trachomatous scarring in Gambians. *BMC Med. Genet.* **10,** 138 (2009).

14. Marti, H., Koschwanez, M., Pesch, T., Blenn, C. & Borel, N. Water-filtered infrared a irradiation in combination with visible light inhibits acute chlamydial infection. *PLoS One* **9,** e102239 (2014).

15. Sela, H. Y. *et al.* Human trophectoderm apposition is regulated by interferon γ-induced protein 10 (IP-10) during early implantation. *Placenta* **34,** 222–230 (2013).

16. Gupta, R., Srivastava, P., Vardhan, H., Salhan, S. & Mittal, A. Host immune responses to chlamydial inclusion membrane proteins B and C in *Chlamydia trachomatis* infected women with or without fertility disorders. *Reprod. Biol. Endocrinol.* **7,** 38 (2009).

17. Aktas, A. *et al.* Higher levels of circulating CXCL-9 and CXCL-11 in euthyroid women with autoimmune thyroiditis and recurrent spontaneous abortions. *Gynecol. Endocrinol.* **30,** 157–60 (2014).

18. Wan, C. *et al.* Progesterone activates multiple innate immune pathways in Chlamydia trachomatis-infected endocervical cells. *Am. J. Reprod. Immunol.* **71,** 165–77 (2014).

19. Lobo, S. C. *et al.* The immune environment in human endometrium during the window of implantation. *Am. J. Reprod. Immunol.* **52,** 244–51 (2004).

20. Molestina, R. E., Miller, R. D., Lentsch, a B., Ramirez, J. a & Summersgill, J. T. Requirement for NF-kappaB in transcriptional activation of monocyte chemotactic protein 1 by *Chlamydia pneumoniae* in human endothelial cells. *Infect. Immun.* **68,** 4282–8 (2000).

21. Shibahara, H. *et al.* Chemokine bioactivity of RANTES is elevated in the sera of infertile women with past *Chlamydia trachomatis* infection. *Am.J Reprod.Immunol.* **49,** 169–173 (2003).

22. Freier, C. P. *et al.* Expression of CCL22 and Infiltration by Regulatory T Cells are Increased in the Decidua of Human Miscarriage Placentas. *Am. J. Reprod. Immunol.* 216–227 (2015). doi:10.1111/aji.12399

23. Srivastava, A., Sengupta, J., Kriplani, A., Roy, K. K. & Ghosh, D. Profiles of cytokines secreted by isolated human endometrial cells under the influence of chorionic gonadotropin during the window of embryo implantation. *Reprod. Biol. Endocrinol.* **11,** 116 (2013).

24. Maxion, H. K. & Kelly, K. A. Chemokine expression patterns differ within anatomically distinct regions of the genital tract during *Chlamydia trachomatis* infection. *Infect. Immun.* **70,** 1538–46 (2002).

25. Porcella, S. F. *et al.* Transcriptional profiling of human epithelial cells infected with plasmid-bearing and plasmid-deficient *Chlamydia trachomatis*. *Infect. Immun.* **83,** 534–43 (2015).

26. Zhou, W.-H., Du, M.-R., Dong, L., Yu, J. & Li, D.-J. Chemokine CXCL12 promotes the cross-talk between trophoblasts and decidual stromal cells in human first-trimester pregnancy. *Hum. Reprod.* **23,** 2669–79 (2008).

27. Hanna, J. *et al.* CXCL12 expression by invasive trophoblasts induces the specific migration of CD16- human natural killer cells. *Blood* **102,** 1569–1577 (2003).

28. Warner, J. A. *et al.* Human cytomegalovirus infection inhibits CXCL12-mediated migration and invasion of human extravillous cytotrophoblasts. *Virol. J.* **9,** 255 (2012).

29. Li, C. M., Hou, L., Zhang, H. & Zhang, W. Y. CCL17 Induces Trophoblast Migration and Invasion by Regulating Matrix Metalloproteinase and Integrin Expression in Human First-Trimester Placenta. *Reprod. Sci.* (2014).
